# Photocatalytic Degradation of Organic Compounds on TiO_2_-Photocatalyst-Coated Concrete Surfaces

**DOI:** 10.3390/molecules30244698

**Published:** 2025-12-08

**Authors:** Katarzyna Bednarczyk, Artur Lewandowski

**Affiliations:** Department of Occupational Safety Engineering, Faculty of Process and Environmental Engineering, Lodz University of Technology, Wolczanska 213 Str., 90-924 Lodz, Poland

**Keywords:** photocatalysis, TiO_2_, concrete cube, self-cleaning surface, water absorption resistance

## Abstract

This study investigates the photocatalytic degradation of organic compounds on TiO_2_-coated concrete paving cubes, with a focus on their potential for environmental remediation in urban settings. The TiO_2_ P25 coating significantly enhanced the photocatalytic activity of the concrete surface, enabling effective degradation of model pollutants such as methylene blue. Various application methods were evaluated, including surface coating with and without impregnation, and bulk incorporation of TiO_2_ into the concrete matrix. Surface properties were assessed using contact angle measurements and absorption tests. Among all tested variants, the surface-coated and impregnated sample (SURF-IMP) showed the highest photocatalytic efficiency, achieving over 67% pollutant degradation. This variant also demonstrated the lowest water absorption and the highest contact angle, confirming improved surface hydrophobicity. In contrast, the bulk-modified sample (MIX) exhibited weaker performance due to limited surface accessibility of TiO_2_ particles. These findings highlight the importance of the application method in optimizing the performance of TiO_2_-functionalized concrete. The developed system offers a practical approach to integrating photocatalytic properties into paving materials for applications such as air purification, surface decontamination, and sustainable urban infrastructure.

## 1. Introduction

The increasing levels of air pollution and environmental degradation in urban areas pose serious challenges to sustainable development, human health, and the longevity of civil infrastructure [1,2,3]. Urban surfaces such as pavements, façades, and concrete structures tend to accumulate particulate matter, organic pollutants, and microorganisms, resulting not only in aesthetic deterioration but also in accelerated chemical and physical degradation [2,4,5]. Addressing these challenges requires innovative multifunctional materials that combine structural performance with environmental self-cleaning and pollutant degradation capabilities [6,7,8].

In this context, photocatalytic materials—particularly titanium dioxide (TiO_2_)—have emerged as promising candidates due to their stability, non-toxicity, abundance, and strong oxidative potential under light irradiation [1,9,10,11]. When exposed to ultraviolet (UV) or visible light, TiO_2_ generates reactive oxygen species (ROS) capable of decomposing a wide range of organic and inorganic contaminants, including volatile organic compounds (VOCs), nitrogen oxides (NO_x_), and hydrocarbons [4,10,11,12,13]. This photocatalytic mechanism has been successfully applied in environmental cleanup, air purification, and wastewater treatment processes [7,14,15,16,17]. In addition, TiO_2_-based materials have shown potential in energy-related applications, such as hydrogen production from organic feedstocks, highlighting their versatility in pollutant degradation and resource transformation [18]. Recent advancements in heterogeneous photocatalysis have significantly improved the design and efficiency of TiO_2_-based materials. Modifications such as metal and non-metal doping (e.g., nitrogen, sulfur, carbon), surface sensitization, and hybrid composite formation have extended TiO_2_ activity into the visible light spectrum, which is crucial for real-world applications where UV radiation is limited [9,14,19,20,21]. Moreover, innovative deposition techniques such as sol–gel processing, spray and dip coating, plasma treatment, and nanostructured film formation have enhanced TiO_2_ adhesion, dispersion, and long-term durability on construction surfaces [22,23,24,25]. These developments have positioned TiO_2_ as a cornerstone material in the emerging field of photocatalytic construction composites, where functionality extends beyond mechanical strength to include environmental remediation and sustainability [12,26,27,28,29].

Concrete, as the most widely used construction material worldwide, provides an ideal substrate for TiO_2_ integration due to its abundance, surface exposure, and structural versatility [19,23,30]. However, its inherently porous microstructure promotes the accumulation of dust, smog particles, and organic pollutants, leading to discoloration, surface weakening, and reduced service life [24,31,32]. The incorporation of TiO_2_ into concrete—either as a surface coating or a bulk additive—offers a viable solution to mitigate these effects. Upon illumination, TiO_2_ promotes photocatalytic degradation of pollutants, thus reducing maintenance needs, improving hydrophilicity and self-cleaning properties, and extending the lifespan of concrete elements [2,7,12,33]. Furthermore, TiO_2_-based coatings have been shown to improve the depolluting efficiency of urban infrastructure by reducing atmospheric concentrations of NO_x_, CO, and VOCs in both indoor and outdoor environments [13,18,34,35,36].

Recent studies have expanded the functionality of TiO_2_-modified construction materials. Nitrogen-doped TiO_2_ composites have shown improved visible-light activity, enabling efficient photocatalytic performance even in shaded or low-light urban environments [10,14]. Hybrid systems such as TiO_2_/SiO_2_, Cu–TiO_2_/SiO_2_, and black TiO_2_ have been developed to enhance light absorption, charge carrier separation, and overall photocatalytic stability [24,25]. Investigations by Khannyra et al. [17] demonstrated that appropriately engineered TiO_2_ coatings retain high self-cleaning efficiency and mechanical integrity even after long-term exposure to environmental stressors. Moreover, research by Kalinowski et al. [33] and Jenim et al. [12] highlighted the potential of nano-TiO_2_-modified cementitious composites to achieve carbon-negative performance through simultaneous air purification and CO_2_ reduction. These findings emphasize the dual environmental and structural benefits of TiO_2_ integration in cementitious materials.

In addition to environmental reactivity, TiO_2_ incorporation improves mechanical behavior and durability of cementitious composites. Studies indicate that optimal nano-TiO_2_ dosages (1–6 wt.%) increase compressive and flexural strength, refine pore structure, and enhance resistance to freeze–thaw cycles and abrasion [34,35,36]. When combined with recycled aggregates such as reclaimed asphalt pavement, CRT glass, or industrial by-products, TiO_2_ improves interfacial bonding while preserving photocatalytic activity, contributing to more sustainable composite design [29,30,32]. Recent research highlights the development of TiO_2_ enhanced building materials for environmental purification [37] and demonstrates the effectiveness of hydrophilic polymer dispersions that improve TiO_2_ dispersion uniformity, resulting in enhanced surface activation and NOx degradation [38], while comparative analyses of spray- and dip-coated layers underscore the importance of the deposition method for long-term self-cleaning performance [39]. Additional advances include immobilization strategies for TiO_2_ on reusable substrates and reactor systems that enhance photocatalytic efficiency and enable catalyst recovery [40], as well as progress in visible-light-activated TiO_2_ materials relevant to pollutant degradation in alkaline environments typical of cementitious systems [41]. Based on these developments, the present study investigates TiO_2_-coated concrete paving units intended for environmental remediation and long-term durability in urban conditions. The experimental program encompasses photocatalytic degradation of organic contaminants and NO_x_ [6,10,14], surface self-cleaning ability [17,24], and changes in surface wettability associated with TiO_2_ coating and dispersion mechanisms [38,39]. Measurements of water and oil absorption, contact angles, and photodegradation efficiency are conducted under natural and artificial illumination, while UV exposure, moisture cycling, and abrasion tests follow methodologies used in durability studies of photocatalytic building materials [12,19,25]. These analyses provide a comprehensive understanding of the potential of TiO_2_-modified concrete to enhance air quality, surface functionality, and material lifespan within urban infrastructure, contributing to the development of sustainable, multifunctional construction materials.

## 2. Materials and Methods

### 2.1. Materials and Chemicals

All reagents used in this study were of analytical grade and applied without further purification. Deionized water was employed for all solution preparations. Concrete paving blocks were fabricated using ordinary Portland cement (CEM I 42.5R), industrial sand (0–2 mm), and gravel (2–8 mm), following standard construction practice to ensure reproducibility and mechanical stability. The detailed composition of the concrete mixture is presented in Table 1.

Concrete cubes with dimensions of 5 × 5 × 5 cm^3^ were cast in steel molds and cured under controlled laboratory conditions (18 ± 2 °C, relative humidity > 90%) for 14 days. The impregnation agent (Feda, Warsaw, Poland) was primarily composed of Tetrahydro-1,3,4,6-tetrakis(hydroxymethyl)imidazo[4,5-d]imidazole-2,5(1H,3H)-dione, stabilized with a mixture of 5-chloro-2-methyl-4-isothiazolin-3-one and 2-methyl-4-isothiazolin-3-one (3:1). Titanium dioxide (TiO_2_, anatase phase, <100 nm, Sigma-Aldrich, Saint Louis, MO, USA) was used as the photocatalyst.

The impregnation system was designed to utilize the four hydroxymethyl (–CH_2_OH) groups in Feda compound, which form hydrogen bonds with hydroxyl groups on the TiO_2_ surface. These interactions enhance the adhesion of nanoparticles to the concrete matrix and limit their potential leaching under aqueous conditions.

### 2.2. Preparation of TiO_2_-Coated Concrete Cubes

The preparation of TiO_2_-coated concrete cubes followed a multi-step procedure to ensure uniform coating and reproducible photocatalytic performance. Initially, dry components—cement, sand, and gravel—were thoroughly mixed until a homogeneous composition was achieved. Deionized water was then gradually added while mixing to reach a workable consistency. The fresh mixture was poured into molds (5 × 5 × 5 cm^3^), compacted to remove air voids, and leveled to achieve surface uniformity. After casting, the molds were placed in a curing chamber maintained at 18 ± 2 °C and relative humidity above 90% for 14 days, ensuring proper hydration and strength development.

**Figure 1:**
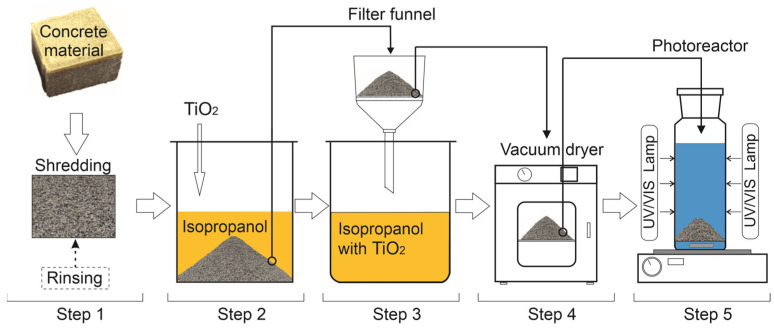
Schematic illustration of concrete cube preparation and TiO_2_ impregnation/coating process. Concrete cubes were cast, cured, and then impregnated and coated with TiO_2_ for uniform surface coverage.

Following the curing process, the TiO_2_/Feda coating was applied via immersion. TiO_2_ nanoparticles were dispersed in deionized water containing 2 wt.% of Feda agent and magnetically stirred for 1 h to ensure complete homogenization. The coating process is schematically illustrated in Figure 1.

The cured concrete cubes were immersed in this dispersion for 10 min, drained, and dried at 60 °C for 2 h. This procedure was repeated up to three times to obtain multilayer coverage, providing enhanced surface uniformity and photocatalytic performance. After coating, the samples were stored under ambient conditions for 24 h before testing. The parameters applied during the coating process are summarized in Table 2.

### 2.3. Analysis of Water Absorption and Wettability

**Figure 2:**
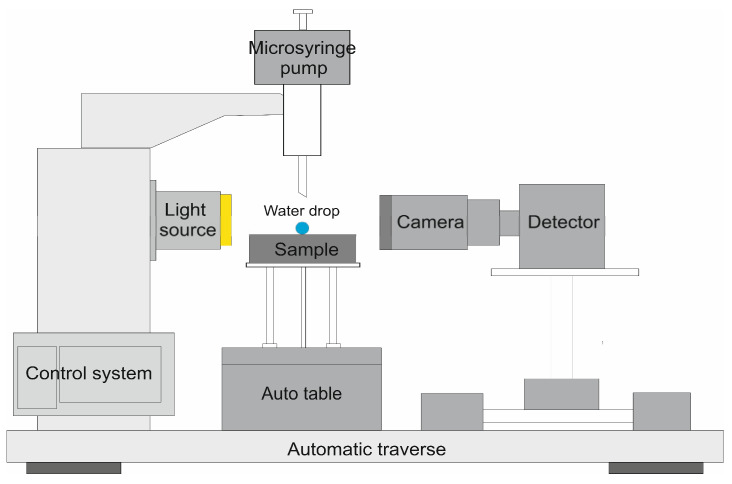
Experimental setup for gravimetric analysis of water absorption using PE foam contact method.

The water absorption behavior of the cementitious samples was analyzed using a gravimetric method based on indirect contact. Prior to testing, all concrete cubes (both TiO_2_/Feda-coated and uncoated reference samples) were dried in a laboratory oven at 105 °C for 3 h to remove physically adsorbed moisture. Each cube was subsequently placed on a polyethylene (PE) foam pad saturated with deionized water, ensuring uniform water delivery to the sample surface (Figure 2). The mass of each sample was recorded at regular intervals using a standard analytical balance (accuracy ±0.1 mg). This setup is distinct from a contact angle measurement, which assesses surface wettability rather than absorption kinetics.

The wetting rate Nw was calculated according to Equation (1):

(1) $$Nw\;=\;\frac{\Delta m}{A\cdot \Delta t}$$

where:

Nw—wetting rate [g/(m^2^·h)];
Δm = m_t_ − m_0_—difference in the mass of the sample at time t(m_t_) and the initial mass (m_0_) [g];
A—surface area of the sample in contact with the sponge, through which absorption occurs [m^2^];
Δt—time interval during which absorption is measured [h].

Contact angle measurements were carried out using a Goniometer OCA 25 (DataPhysics Instruments, Filderstadt, Germany) to determine surface wettability (10 μL water droplets). Microscopic observations were performed using a Bresser Researcher Bino Binocular Microscope 40×–1000× (Bresser, Rhede, Germany) to examine surface morphology and coating uniformity. The oven drying parameters were 105 °C for 3 h.

This experimental setup allows reproducible evaluation of water absorption kinetics and wettability of both TiO_2_/Feda-coated and uncoated concrete samples, providing the basis for subsequent quantitative analysis in the Results and Discussion section.

### 2.4. Mechanical Properties of TiO_2_-Modified Concrete Cubes

The mechanical properties of the concrete cubes were evaluated using compressive strength testing. Concrete specimens with dimensions of 50 × 50 × 40 mm were prepared and classified into four groups:

- REF—reference concrete cube without TiO_2_;
- SURF-noIMP—concrete cube with TiO_2_ (P25) applied on the surface, without impregnation;
- SURF-IMP—concrete cube with TiO_2_ (P25) applied on the surface with impregnation;
- MIX—concrete cube with TiO_2_ (P25) distributed throughout the concrete mass (0.5 wt.%).

All samples were cured for 28 days under humid conditions. Prior to testing, the specimens were dried to constant mass. Compressive strength measurements were conducted using a MEGA 100 universal testing machine (ViaTeco, Warszawa, Poland). For each sample type, three replicates (*n* = 3) were tested, and the mean values with standard deviations (SD) were reported.

### 2.5. Photocatalytic Activity Tests

**Figure 3:**
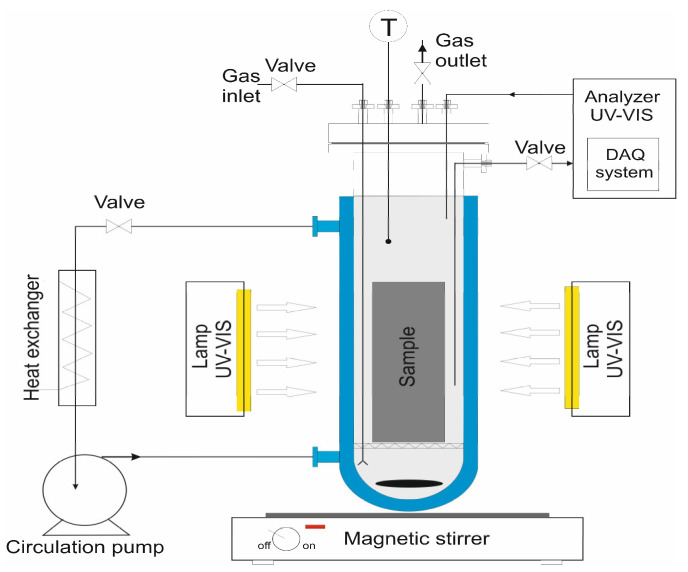
Schematic diagram of the photocatalytic reactor (75 W × 4 Xe-arc; Shredded paving stone with TiO_2_ and methylene blue dye addition).

The photocatalytic performance of TiO_2_-coated concrete cubes was evaluated using methylene blue (MB) as a model organic pollutant. Each concrete cube was uniformly coated with a 10 mg/L aqueous solution of MB and allowed to rest in the dark for 1 h to reach adsorption–desorption equilibrium. Subsequently, the samples were irradiated under a Xe-arc lamp (4 × 75 W, spectral range 320–780 nm, light intensity 55 W/m^2^ at a distance of 20 cm) for 4 h. The experimental setup for the photocatalytic tests is presented in Figure 3.

The concentration of residual methylene blue (MB) was measured spectrophotometrically at 664 nm, the wavelength of maximum absorbance of the dye. All measurements were performed in triplicate (*n* = 3) to ensure reproducibility. The main experimental parameters for the photocatalytic degradation tests, including TiO_2_ loading, sample geometry, irradiation conditions, and solution volume, are summarized in Table 3. The photocatalytic degradation process was monitored by recording changes in MB absorbance at regular time intervals throughout the irradiation period.

## 3. Results and Discussion

### 3.1. Effect of Surface TiO_2_ Loading (ppm) on the Preparation of Concrete Cubes

Preliminary optimization tests were conducted to evaluate the influence of TiO_2_ concentration and its mode of application on the physicochemical properties of concrete paving cubes. TiO_2_ suspensions in isopropanol were prepared at varying concentrations and applied either onto the surface or incorporated within the bulk material. Among the tested concentrations, 5000 ppm exhibited the most stable dispersion, homogeneous deposition, and reproducible photocatalytic performance under laboratory conditions [11,33].

**Figure 4:**
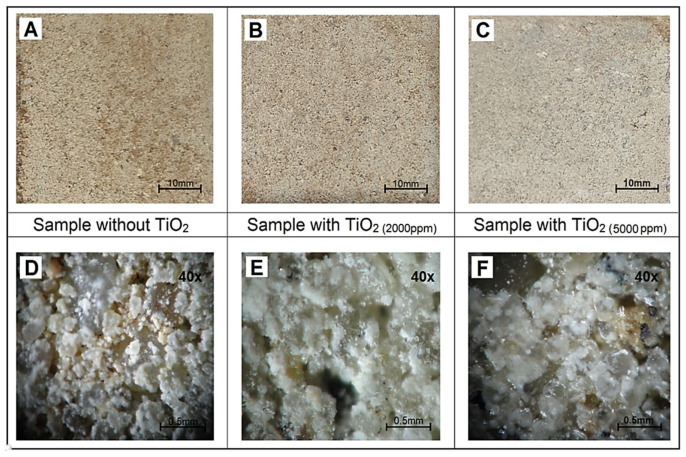
Paving stones without and with the addition of TiO_2_: (**A**–**C**) Surface images showing differences in appearance between the reference sample and the sample with TiO_2_; (**D**–**F**) Microscopic views (40×) illustrating changes in surface texture resulting from the addition of TiO_2_.

As illustrated in Figure 4, the uncoated reference sample (REF) presented a heterogeneous concrete surface with open pores and a rough morphology, which facilitates water ingress but provides limited chemical activity. At 2000 ppm TiO_2_, partial surface coverage was observed, indicating initial photocatalyst adhesion, although the distribution remained non-uniform. Increasing the loading to 5000 ppm produced a darker and smoother surface, suggesting the formation of a continuous TiO_2_ layer that enhances light absorption and active-site availability, in line with observations by Khannyra et al. [17] and Luna et al. [25]. Therefore, subsequent experiments were performed using 5000 ppm TiO_2_ as the optimized surface concentration.

### 3.2. Wettability and Water Absorption Behavior

**Figure 5:**
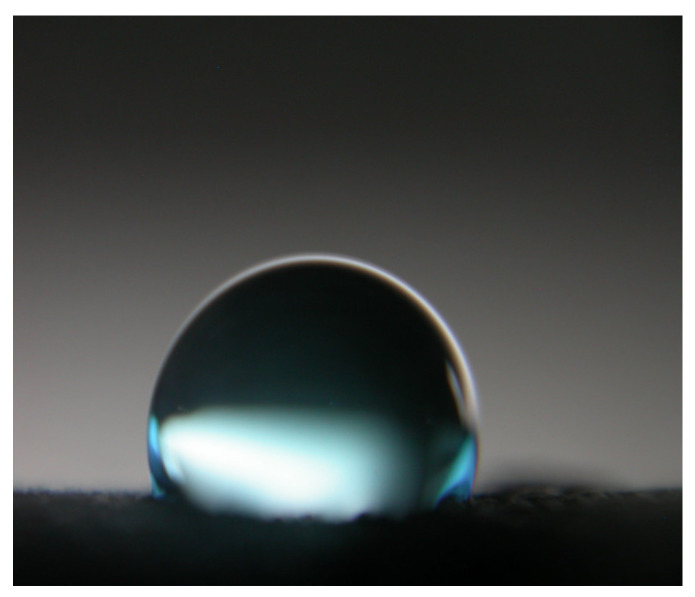
Image of a distilled water droplet placed on the SURF-IMP concrete surface during static contact angle measurement (droplet volume: 10 µL, temp.: 23 ± 1 °C, RH (relative humidity): 50 ± 5%, measurement time after droplet deposition: 5 s).

The influence of TiO_2_ surface modification on the wettability and water absorption behavior of concrete paving cubes was evaluated. Contact angle (CA) measurements revealed a significant alteration in surface energy due to the presence of TiO_2_ coatings (Figure 5, Table 4). The unmodified reference concrete (REF) exhibited complete wetting (CA ≈ 0° for both water and oil), confirming its inherently hydrophilic and oleophilic character.

Among the TiO_2_-modified samples, SURF-noIMP (TiO_2_ deposited on the surface without impregnation) displayed moderate water (77.1°) and oil (31.7°) contact angles, indicating partial resistance to liquid spreading. The MIX variant, where TiO_2_ nanoparticles were incorporated into the bulk concrete, showed lower contact angles (67.2° for water; 19.4° for oil), reflecting limited availability of active TiO_2_ at the surface. In contrast, the SURF-IMP sample, subjected to surface impregnation, exhibited the highest hydrophobic and oleophobic response (water CA = 86.5°, oil CA = 37.1°), highlighting the effect of the impregnation process in densifying the near-surface layer and promoting chemical anchoring of TiO_2_ nanoparticles. This is consistent with prior studies reporting enhanced surface performance through impregnation strategies [24,38].

The water absorption kinetics, evaluated via gravimetric analysis using saturated PE foam pads, further supported these observations. Reference samples (R) absorbed water rapidly (Nw = 80 g/m^2^·h), whereas TiO_2_-coated specimens showed significantly reduced water uptake (Table 5). The SURF-IMP sample demonstrated the lowest wetting rate (Nw = 32 g/m^2^·h), indicating that the combination of TiO_2_ deposition and surface impregnation effectively reduces capillary absorption, improving the durability and functional performance of concrete surfaces.

These results collectively demonstrate that TiO_2_ coatings reduce water uptake and modify surface energy, with the impregnation method yielding the most pronounced effects. The observed trends are in agreement with previous reports indicating that surface-exposed TiO_2_ enhances hydrophobicity and self-cleaning potential, while bulk-embedded particles contribute less to surface functionalization [8,17,24,38].

Microscopic observations using a Bresser Researcher Bino Binocular Microscope (Bresser, Germany) revealed uniform coverage in the SURF-IMP samples, whereas MIX and SURF-noIMP surfaces exhibited partial or non-uniform TiO_2_ distribution. Future work will employ SEM/EDS analysis to investigate nanoparticle distribution, adhesion, and interactions within the cementitious matrix at the micro- and nanoscale.

**Figure 6:**
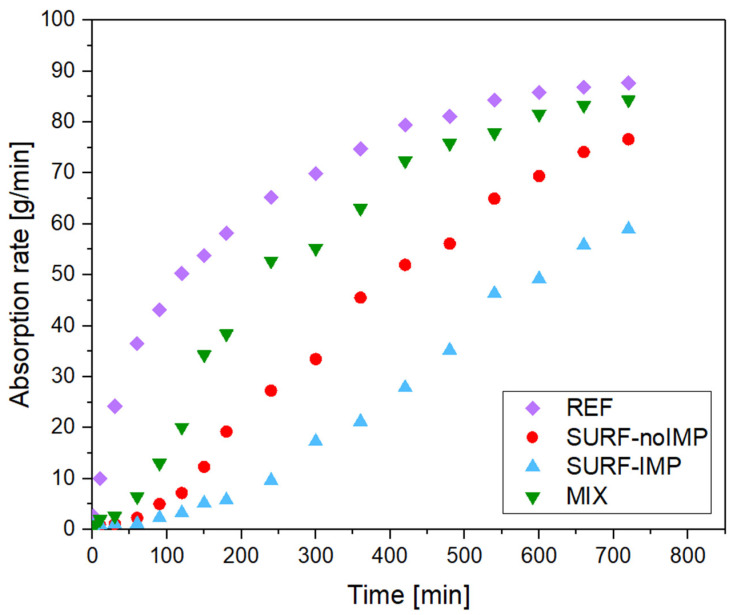
Water absorption rate of concrete cubes prepared using different TiO_2_ application methods: SURF-noIMP (TiO_2_ applied on the surface without impregnation), SURF-IMP (TiO_2_ applied on the surface with impregnation), MIX (TiO_2_ distributed throughout the entire concrete mass), and REF (reference sample without TiO_2_).

The correlation between surface chemistry and water absorption is shown in Figure 6. After 720 min of immersion, the REF sample exhibited the highest absorption rate (87.69%), while the SURF-IMP specimen showed the lowest (58.96%). The reduced absorption of SURF-IMP can be attributed to the filling and nucleation effects of TiO_2_, which refine the pore network and strengthen the interfacial transition zone (ITZ) through enhanced C–S–H formation [35,36]. This dual mechanism limits water ingress and supports the development of durable, photocatalytically active surfaces suitable for long-term urban exposure.

### 3.3. Compressive Strength of Concrete Cubes

The prepared concrete cubes containing TiO_2_ exhibited enhanced technical properties, including higher compressive strength and lower water absorption, while maintaining favorable mechanical performance. The compressive strength results of the different concrete samples after 28 days of curing under moist conditions are summarized in Table 6.

Analysis of the data presented in Table 6 shows that the SURF-noIMP and SURF-IMP samples, containing approximately 5.9 g TiO_2_/m^2^ on the surface and within surface pores, exhibited no significant change in compressive strength compared with the reference sample (REF), with mean values of 42.1 MPa. The surface impregnation effectively minimized TiO_2_ leaching from the concrete surface, while maintaining the mechanical integrity of the material.

In contrast, the MIX samples, with TiO_2_ homogeneously distributed throughout the concrete matrix, showed an increase in compressive strength relative to REF. Incorporation of 5.0 g TiO_2_ per kg of concrete (0.5 wt.%) led to an increase in compressive strength from 41.6 to 44.3 MPa, corresponding to an improvement of approximately 6.5%. This enhancement is attributed to the micro-filling effect of the nanoparticles. The addition of 0.5 wt.% nano-TiO_2_ promotes cement hydration through a nucleation effect, resulting in a denser microstructure and reduced porosity compared with concrete samples without TiO_2_.

### 3.4. Photocatalytic Degradation of Pollutants

**Figure 7:**
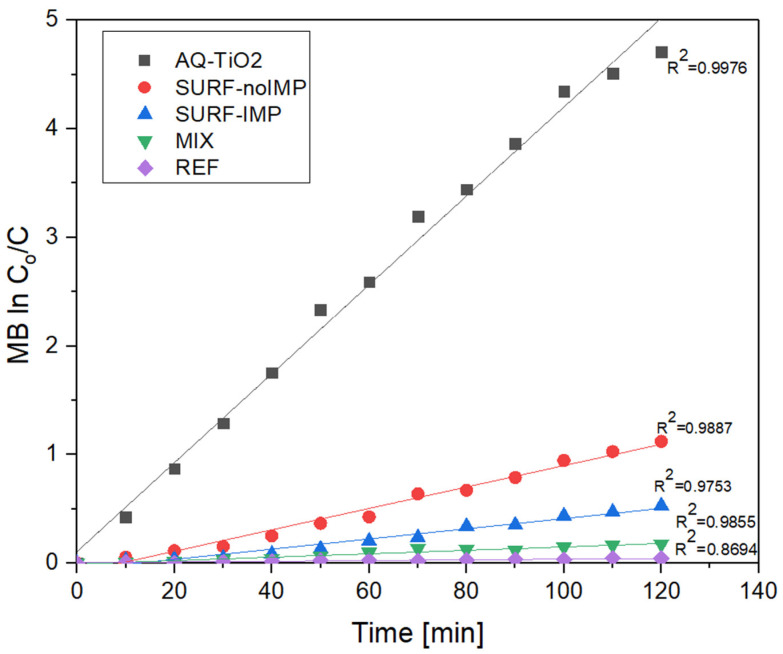
Photocatalytic degradation of methylene blue (MB) on concrete samples over 120 min: AQ-TiO_2_ (aqueous TiO_2_ suspension), SURF-noIMP (TiO_2_ on surface without impregnation), SURF-IMP (TiO_2_ on surface with impregnation), MIX (TiO_2_ distributed throughout concrete mass), and REF (reference without TiO_2_).

The photocatalytic degradation efficiency of methylene blue (MB) was evaluated over 120 min under simulated solar irradiation (Figure 7). The quantitative results, including initial and final molar concentrations and calculated degradation degrees with standard deviations, are summarized in Table 6. These data confirm that TiO_2_-modified surfaces exhibit markedly improved pollutant removal compared with the uncoated reference sample.

The reference sample (REF) showed minimal degradation (4.11%), confirming the absence of any photocatalytic contribution from the unmodified concrete. In contrast, all TiO_2_-coated samples exhibited significant degradation, proportional to the degree of TiO_2_ surface exposure. The SURF-noIMP sample achieved a degradation rate of 41.11%, reflecting the inherent photocatalytic activity of surface-deposited TiO_2_. The MIX sample, with TiO_2_ uniformly distributed throughout the concrete matrix, displayed only 15.99% degradation, as the majority of nanoparticles remained embedded within the cement paste and were shielded from light and reactants. This behavior is consistent with previous studies showing that only exposed TiO_2_ surfaces contribute to photochemical reactions [13,20,31].

The SURF-IMP configuration exhibited the highest photocatalytic efficiency (67.41%), highlighting the synergistic effect of the impregnation process. The functional impregnation medium, containing hydroxymethyl-rich imidazolidinone derivatives, likely promoted TiO_2_ particle retention through hydrogen bonding and covalent Si–O–Ti linkages, preventing photocatalyst leaching and sustaining long-term reactivity [25,33,38]. The improved surface uniformity and compactness enhance light absorption and active-site availability, directly contributing to the increased degradation kinetics of methylene blue.

The photocatalytic performance of concrete cubes with different TiO_2_ surface treatments was evaluated based on the degradation of methylene blue. Table 7 presents the initial and final molar concentrations of the pollutant along with the degree of degradation for each sample. The SURF-IMP configuration demonstrated the highest photocatalytic efficiency (67.41%), followed by SURF-noIMP (41.11%) and MIX (15.99%), while the uncoated reference (REF) exhibited negligible degradation (4.11%). The low variability between replicates (SD ≤ 3.5%) confirms the reproducibility of the measurements.

The superior efficiency of the SURF-IMP samples is attributed to the functional impregnation, which enhances TiO_2_ retention and maintains an active surface layer through hydrogen bonding and covalent Si–O–Ti interactions [38]. In contrast, TiO_2_ dispersed within the bulk (MIX) shows lower surface availability, resulting in reduced photocatalytic activity [33]. The effectiveness of surface-localized TiO_2_ in promoting pollutant degradation and self-cleaning performance is consistent with previous studies on cementitious materials [9,24]. Overall, the results indicate that optimized surface loading and stable retention of TiO_2_ significantly enhance the photocatalytic performance of concrete paving blocks, in line with general principles of heterogeneous photocatalysis [3].

## 4. Conclusions

The results of this study demonstrate that the method use for TiO_2_ incorporation exerts a decisive influence on both the photocatalytic efficiency and surface performance of concrete paving blocks. Among all tested configurations, the surface application of TiO_2_ combined with a functional impregnation treatment proved to be the most effective modification strategy. This system achieved the highest pollutant degradation efficiency—over sixteen times greater than that of the unmodified reference sample—confirming the pronounced photocatalytic activity of the treated surface.

In addition to superior photocatalytic performance, the impregnated TiO_2_ coating significantly reduced water absorption and increased the contact angle values, indicating enhanced hydrophobicity and improved resistance to environmental factors such as moisture ingress and surface fouling. These characteristics are essential for extending the service life, self-cleaning capability, and long-term durability of concrete elements exposed to outdoor conditions. Conversely, the incorporation of TiO_2_ nanoparticles within the bulk of the concrete matrix yielded markedly lower photocatalytic activity. This is primarily attributed to the partial encapsulation of TiO_2_ particles within the cementitious microstructure, which limits their accessibility to ultraviolet radiation and atmospheric pollutants. Consequently, the spatial distribution and stabilization of TiO_2_ on the concrete surface are key determinants of its functional effectiveness.

Overall, the application of TiO_2_ through surface coating combined with impregnation presents a practical and scalable approach for producing multifunctional, photocatalytically active concrete materials. Such surfaces not only enhance the environmental performance of urban infrastructure—by contributing to air purification and self-cleaning effects—but also improve durability and reduce maintenance demands. Therefore, this technology represents a promising pathway toward the development of sustainable and intelligent construction materials for next-generation urban environments.

## Figures and Tables

**Table 1:** Composition of the concrete mixture.

Component	Parts (by Weight)	Remarks
Portland cement	1	Type 42.5R
Sand	2.5	Industrial, 0–2 mm
Gravel	4.5	2–8 mm
Water	0.45	w/c = 0.45
Curing temperature	18 ± 2 °C	Humidity > 90%
Curing time	14 days	Standard hydration

**Table 2:** TiO_2_ coating parameters.

Parameter	Value
TiO_2_ type	Anatase, <100 nm
Dispersion medium	Deionized water + 2 wt.% Feda agent
Immersion time	10 min
Drying temperature	60 °C
Drying duration	2 h
Number of layers	1–3
Post-curing time	24 h

**Table 3:** Photocatalytic degradation test parameters.

Parameter	Value
Light source	Xe-arc lamp, 75 W × 4
Spectral range	320–780 nm
Light intensity	55 W/m^2^ at sample surface
Distance	20 cm
Exposure time	4 h
Pollutant	Methylene blue, 10 mg/L
Detection wavelength	664 nm
Replicates (*n*)	3

**Table 4:** Wettability Analysis of Cubic Concrete Samples Using the Contact Angle Method.

Name of the Tested Sample		Test Substances	
		Distilled Water	Oil
		Wet Angle of Concrete Cube α [°]	
Sample	REF	no angle	no angle
without TiO_2_			
Sample with TiO_2_	SURF-noIMP (5000 ppm TiO_2_ outside)	77.1	31.74
	MIX (5000 ppm TiO_2_ inside)	67.2	19.4
	SURF-IMP (5000 ppm TiO_2_ outside and concrete impregnation)	86.5	37.1

**Table 5:** Example results of water absorption.

Sample	TiO_2_ Coating	Initial Mass m_0_ (g)	Mass After t (g)	Nw [g/m^2^·h]
R1	No	50.0	52.0	80
T1	Yes	50.0	51.0	40
T2	Yes	50.0	50.8	36

**Table 6:** Compressive strength of concrete cubes (mean ± SD, *n* = 3).

Name of the Tested Sample		TiO_2_ Application Method	Compressive Strength (MPa) for 14 Day
REF	Reference concrete cube	Without TiO_2_,	41.6 ± 1.7
SURF-noIMP	(5000 ppm TiO_2_ outside)	On the surface of concrete	42.1 ± 2.3
SURF-IMP	(5000 ppm TiO_2_ outside and concrete impregnation)	On the surface of concrete	42.1 ± 1.9
MIX	5000 ppm TiO_2_ inside	In the entire volume of the concrete cube	44.3 ± 2.1

**Table 7:** Initial and final molar concentrations of pollutant and degree of degradation (SD) on concrete cubes with and without TiO_2_ coating.

Name of Sample	Initial Molar Concentration (Before Reaction) C_o_	Final Molar Concentration (After Reaction) C_k_	Degree of Degradation SD
	[mol/dm^3^]	[mol/dm^3^]	[%]
REF	4.56 × 10^−5^	4.37 × 10^−5^	4.11
SURF-noIMP	4.56 × 10^−5^	2.68 × 10^−5^	41.11
MIX	4.56 × 10^−5^	3.83 × 10^−5^	15.99
SURF-IMP	4.56 × 10^−5^	1.49 × 10^−5^	67.41

## Data Availability

The original contributions presented in this study are included in the article. Further inquiries can be directed to the corresponding authors.

## Abbreviations

The following abbreviations are used in this manuscript:

AQ-TiO_2_	aqueous solution containing TiO_2_ (P25)
SURF-noIMP	concrete cube with TiO_2_ (P25) on the surface, no impregnation
SURF-IMP	concrete cube with TiO_2_ (P25) on the surface, with impregnation
MIX	concrete cube with TiO_2_ (P25) distributed throughout the entire concrete mass
REF	reference concrete cube without TiO_2_
Xe-arc	xenon lamp
